# Quantitative MRI methods for the assessment of structure, composition, and function of musculoskeletal tissues in basic research and preclinical applications

**DOI:** 10.1007/s10334-024-01174-7

**Published:** 2024-06-21

**Authors:** Victor Casula, Abdul Wahed Kajabi

**Affiliations:** 1 Research Unit of Health Sciences and Technology, Faculty of Medicine, University of Oulu, Oulu, Finland; 2 Center for Magnetic Resonance Research, University of Minnesota, Minneapolis, MN USA; 3 Department of Radiology, University of Minnesota, Minneapolis, MN USA

**Keywords:** Magnetic resonance imaging, Osteoarthritis, Cartilage, Meniscus, Muscles

## Abstract

Osteoarthritis (OA) is a disabling chronic disease involving the gradual degradation of joint structures causing pain and dysfunction. Magnetic resonance imaging (MRI) has been widely used as a non-invasive tool for assessing OA-related changes. While anatomical MRI is limited to the morphological assessment of the joint structures, quantitative MRI (qMRI) allows for the measurement of biophysical properties of the tissues at the molecular level. Quantitative MRI techniques have been employed to characterize tissues’ structural integrity, biochemical content, and mechanical properties. Their applications extend to studying degenerative alterations, early OA detection, and evaluating therapeutic intervention. This article is a review of qMRI techniques for musculoskeletal tissue evaluation, with a particular emphasis on articular cartilage. The goal is to describe the underlying mechanism and primary limitations of the qMRI parameters, their association with the tissue physiological properties and their potential in detecting tissue degeneration leading to the development of OA with a primary focus on basic and preclinical research studies. Additionally, the review highlights some clinical applications of qMRI, discussing the role of texture-based radiomics and machine learning in advancing OA research.

## Introduction

Osteoarthritis (OA) is a multifaceted disorder affecting diarthrodial joints, characterized by the gradual degradation of cartilage, bones, menisci, ligaments, and tendons, accompanied by synovial inflammation arising from diverse biochemical and biomechanical mechanisms [1]. OA stands as one of the most prevalent chronic degenerative diseases, exerting a substantial global burden of disability [2], with its prevalence rising significantly with age and obesity. Projections further suggest an exponential increase in the upcoming decades, placing additional strain on healthcare systems [3]. Presently, disease-modifying drugs remain unavailable, leaving the management of OA confined to symptomatic treatments and joint replacement surgeries in severe cases.

Over the last two decades, various imaging-based methodologies have emerged for early OA detection and monitoring the efficacy of potential therapeutic interventions. Among these, magnetic resonance imaging (MRI) has become a fundamental and widely used tool in preclinical and clinical OA research [4]. While anatomical MRI is frequently employed for the morphological assessment of joint tissues [5], it lacks the capability to detect biochemical and microstructural changes preceding gross morphological alterations. A plethora of quantitative MRI (qMRI) techniques has been proposed, enabling the direct measurement of biophysical tissue properties at the molecular level. Techniques like T2 and T1ρ relaxation time mapping quantify tissues’ magnetic properties to characterize biochemical content, architecture, and functional condition [6, 7]. Other techniques allow for the indirect assessment of specific biochemical components. For instance, delayed gadolinium-enhanced MRI of cartilage (dGEMRIC) [8], sodium (^23^Na) MRI [9], and glycosaminoglycan (GAG) chemical exchange saturation transfer [10] provide insights into GAG content. Quantitative MRI has been extensively employed in collagenous tissues, particularly articular cartilage, to explore composition, structural integrity, and mechanical properties in normal, artificially degenerated cartilage samples, and animal models of OA [11, 12]. Moreover, it has been proven valuable in evaluating cartilage quality in both preclinical and clinical studies [13], as well as after reparative and regenerative surgical treatment of osteochondral lesions [14].

This paper provides an overview of qMRI techniques for musculoskeletal tissue evaluation (Table 1), including their underlying mechanisms, correlations with physiological properties, potential for detecting degeneration, and primary limitations. Notably, several reviews in the literature mostly present qMRI applications in clinical OA research. The primary focus of this review centers on qMRI applications in basic research and preclinical studies, with a particular emphasis on articular cartilage research. As such, a detailed review of clinical research on OA involving qMRI is beyond the scope of this paper, and interested readers can refer to comprehensive review articles available in the literature for further insights [4, 13, 15, 16].

**Table 1:** Summary of established and emerging quantitative MRI techniques for musculoskeletal tissues

Parameter	Tissue	Correlates with	Applications	Interpretation	Strengths and Limitations
T2 mapping	Cartilage	Water content, collagen orientation, collagen content; tissue mechanical properties; OA histological grade	Cartilage integrity and degeneration in animal models, in vitro enzymatic degradation, etc.; cartilage function and response to load; response to treatment; in vivo clinical studies	Increase in T2 relaxation time indicates increase in tissue hydration and a loss of integrity of the extracellular matrix	• Well-established in research and clinical settings • Reveals biochemical and structural changes in cartilage before visible morphological damage • Susceptible to magic angle effect • Dependent on measurement settings and analysis methods
	Menisci	Water and collagen content, collagen organization, GAG content; tissue mechanical properties	Quality and degeneration in animal models, in vitro enzymatic degradation, etc.; tissue function and response to load; in vivo clinical studies	Increase in T2 relaxation time may indicate tissue disorganization associated with meniscal degeneration or injury	• Not fully validated, needs further research • Provides insights into meniscal composition and function • Similar challenges as in cartilage assessment (magic angle, lack of standardization)
	Ligaments and tendons	Histological evaluation; mechanical properties	Tissue healing post-treatment	Not fully clarified	• Not validated, needs further research • Similar challenges as in cartilage assessment (magic angle, lack of standardization)
T2* mapping	Cartilage	Collagen orientation, GAG content; mechanical properties; cartilage degeneration	Quality and degeneration in animal models, in vitro enzymatic degradation, etc.; tissue function and response to load; in vivo clinical studies	Increase in T2* relaxation time may indicate tissue disorganization associated with degeneration	• Well established • Faster than T2 mapping • Less tissue specific compared to T2 and T1ρ • Prone to magnetic susceptibility and magic angle effect
	Menisci	Water and GAG content, collagen content and organization; mechanical properties	Degeneration in ex vivo studies	Increase in T2* relaxation time may indicate tissue disorganization associated with meniscal degeneration or injury	• Not validated, needs further research • Similar limitations as in cartilage assessment
	Ligaments and tendons	Histological evaluation; mechanical properties	Tissue tendon healing	Not fully clarified	• Not validated, needs further research • Similar challenges as in cartilage assessment
	Bone	Bone microstructure; mineral density; mechanical properties	Bone characterization	Not fully clarified	• Not validated, needs further research
T1ρ mapping	Cartilage	Water and GAG content, collagen orientation and content; tissue mechanical properties; OA histological grade	Cartilage integrity and degeneration in animal models, in vitro enzymatic degradation, etc.; cartilage function and response to load; response to treatment; in vivo clinical studies	Increase in T1ρ relaxation time indicates increase in tissue hydration, GAG loss, and a loss of integrity of the extracellular matrix	• Well-established • Reveals cartilage biochemical and structural changes before any visible morphological damage • More sensitive to GAG changes comparted to T2 and T2* • Susceptible to magic angle (less than T2) • Dependent on measurement settings and analysis method
	Menisci	Water and GAG content, collagen content and organization; mechanical properties	Quality and degeneration in ex vivo studies; in vivo clinical studies	Increase in T1ρ relaxation time may indicate meniscal degeneration	• Not validated, needs further research
	Ligaments	Mechanical properties	Mechanical assessment of ligament ex vivo	Not clarified	• Not validated, needs further research
dGEMRIC (T1Gd)	Cartilage	GAG content; mechanical properties	Noninvasive evaluation of GAG content in cartilage; cartilage quality assessment in vivo and ex vivo	Decrease in T1Gd is reflective of decrease in GAG content and worsen biomechanical properties	• Well-established • Specificity for GAG content • Reveals compositional changes before gross morphological alterations • Dependent on the permeability of cartilage, long acquisition time
^23^Na MRI	Cartilage	GAG content	Noninvasive assessment of GAG content in cartilage; evaluates cartilage homeostasis and OA	Decrease in ^23^Na concentration indicates GAG loss and imply cartilage degradation	• Validated • Specificity for GAG content • Low SNR and resolution due to low concentration and rapid signal decay compared to ^1^H MRI • Long scan time
	Tendons	GAG content	Assessment of GAG content ex vivo	Decrease in ^23^Na concentration indicates GAG loss	• Not validated, needs further research • Similar challenges as in cartilage assessment
	Muscles	Intracellular and extracellular sodium ion concentration	Assess muscular disorders such as inflammatory myopathies and muscular dystrophy	Increase in sodium ion concentration reflects histopathological abnormalities	• Not validated, needs further research • Sensitive to sodium concentration and fatty infiltration caused by muscular diseases • Similar challenges as in cartilage assessment
gagCEST	Cartilage	GAG content; mechanical properties	Cartilage degeneration	Increase in gagCEST reveals high GAG content and high load-bearing capacity	• Not extensively validated, needs further research • Specificity for GAG content • Complex post-processing required • Benefit from high magnetic field strengths
Diffusion imaging	Cartilage	Diffusion of water molecules; Water content; GAG/Collagen content	Identification of microstructural changes	Increased ADC values signifies PG depletion, FA reflects collagen fiber orientation	• Not fully validated, needs further research • Sensitive to early cartilage degeneration • Sensitive to PG depletion and collagen fiber network • Visualization of collagen fiber architecture with DTI • Limited resolution • Benefit from high magnetic field strengths
	Menisci, Ligaments, tendons, muscles	Diffusion of water molecules	Identification of microstructural changes	FA reflects collagen fiber orientation	• Not validated, needs further research • Visualization of collagen fiber architecture • Limited resolution • Benefit from higher magnetic field strengths
Quantitative susceptibility mapping (QSM)	Cartilage	Collagen fiber orientation and calcification, GAG content; mechanical properties	Evaluate degenerative changes in collagen fiber network	Changes in magnetic susceptibility may indicate alterations in collagen network	• Not extensively validated, needs further research • Susceptibility to artifacts
	Cortical bone	Mineral density	Assess intracortical bone mineral density	Low values reflect high mineral density	• Not validated, needs further research

## Native relaxometry of articular cartilage

**Fig. 1:**
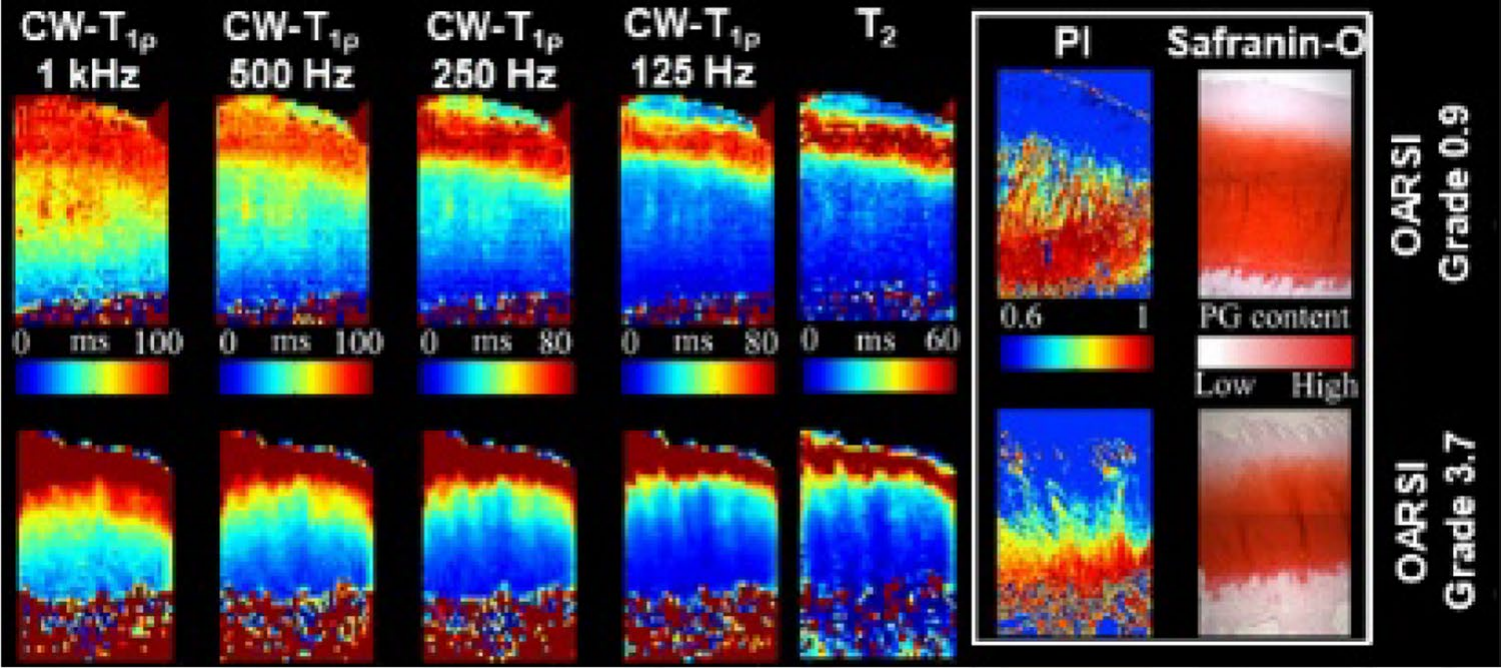
Representative T2 and T1ρ maps and histological images showing collagen fibril anisotropy (parallelism index, PI) and PG content (Safranin-O) of osteochondral specimens obtained from tibial plateaus of patients with early OA (OARSI grade 0.9) and advanced OA (OARSI grade 3.7). Prolonged T2 and T1ρ values are depicted on the maps particularly in the superficial cartilage of the patient with advanced OA, which are consistent with collagen disorganization and PG loss, as depicted by the histology. Tri-laminar appearance of cartilage is demonstrated by the T2 and T1ρ at low spin-locking frequencies (≤ 500 kHz). Adapted from [41] and reproduced with permission of John Wiley & Sons, copyright 2023

**Fig. 2:**
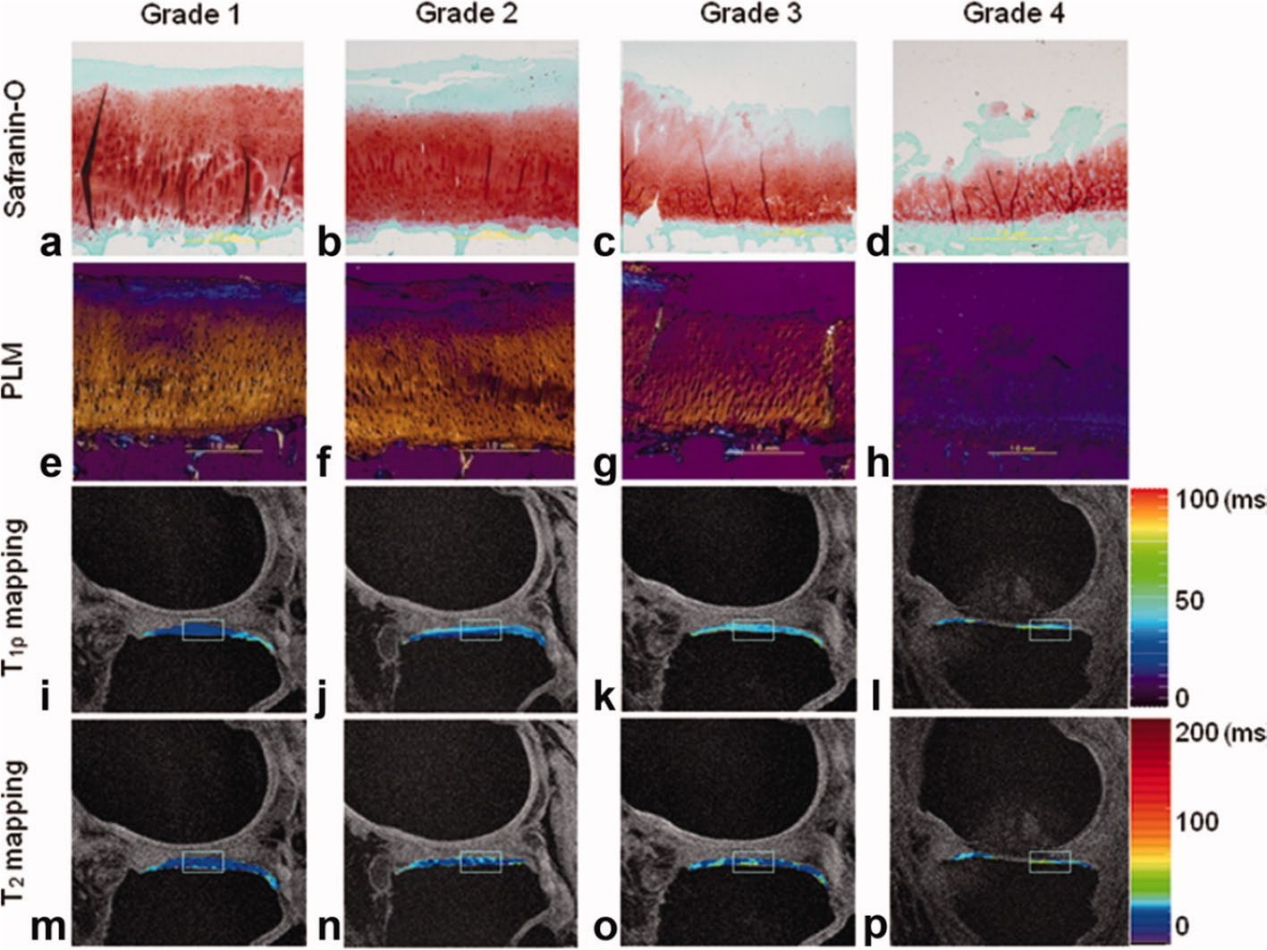
Histological images including optical density (absorbance) of Safranin-O (**a**–**d**) and polarized light microscopy (PLM) (**e**–**h**) of articular cartilage reveal decreasing proteoglycan (PG) content and disrupted collagen fibril orientation, respectively, with increasing OARSI grades. The corresponding regions (indicated as rectangles) on the T1ρ (**i**–**l**) and T2 maps (**m**–**p**) depict elevated relaxation time values, which are consistent with increasing OARSI grades and degenerative changes measured with histology. Reproduced from [23] with permission of John Wiley & Sons, copyright 2023

T2 relaxation time mapping is the most established qMRI technique, extensively investigated in validation studies with chondral samples and animal models of OA. T2 relaxation time mapping quantifies the attenuation of transverse magnetization within the tissue, which is associated with the dipolar interaction of proton spins, and in cartilage is primarily influenced by the restricted motion of water molecules within the extracellular matrix [6]. As a result, T2 is susceptible to molecular and organizational changes in cartilage. Specifically, T2 measurements reflect tissue hydration and provide information on collagen orientation and content. Biochemical assays of ex vivo cartilage have shown that T2 relaxation time correlates positively with water content and negatively with collagen content [17–19]. Furthermore, the spatial variation of T2 is closely associated with the three-dimensional arrangement of the collagen fibrils as observed through polarized light microscopy [20–22]. This enables the visualization of the typical laminar appearance of cartilage in T2 maps, which reveals the orientation of collagen fibers in different histological zones (Fig. 1) [19, 21]. As an increase in the water content and disruption in the organization of the collagen matrix are among the earliest physiologic changes in cartilage degeneration, T2 mapping serves as a valuable tool for detecting subtle OA-related alterations even before conventional morphological images reveal macroscopic lesions. T2 relaxation time has been reported to increase with cartilage degeneration (Figs. 1, 2) particularly with loss of collagen matrix integrity [23, 24]; therefore, T2 mapping enables assessment of OA progression. Additionally, T2 relaxation times have been found to correlate with cartilage mechanical properties, showing a negative correlation with Young’s modulus and dynamic modulus [22, 24–26]. Thus, T2 provides insight into the functional status of the tissue. Several methods have been proposed for T2 mapping [28–31]. The most common approach involves acquiring multiple images with varying echo times using single-echo or multi-echo spin-echo sequences, followed by fitting an exponential function to the signal intensity of these images. Despite its main strength in sensitivity to collagen fiber orientation, the technique also faces significant challenges, as T2 values are dependent on the tissue's orientation with respect to the direction of the main magnetic field [32, 33]. This phenomenon, known as the magic angle effect, makes the interpretation of T2 maps challenging. Additional drawbacks include long scan times and T2 quantitation dependency on the acquisition method, imaging parameters, MRI scanner, and analysis method used, which are common challenges across quantitative MRI techniques.

Another commonly used qMRI technique in OA research is T2* relaxation time mapping, which also serves as an assessment tool for cartilage quality. T2* relaxation is influenced by processes similar to those contributing to T2 relaxation, with an additional impact from the magnetic field inhomogeneity [34]. As local field inhomogeneities reflect tissue structure, T2* mapping has the potential to reveal changes in the cartilage extracellular matrix. Specifically, magnetic susceptibility variations related to collagen fiber alignment within the tissue are captured by T2*, which makes it particularly relevant when imaging highly ordered tissues such as articular cartilage, tendons, or ligaments. Similar to T2, T2* has been reported to be strongly sensitive to collagen orientation and positively correlated with histological grading of cartilage degeneration [33, 35, 36]. Nykänen et al. have reported moderate negative correlations of T2* with GAG content, Young’s modulus, and dynamic modulus [36]. T2* relaxation time can be measured from the exponential decay of the signal using a gradient echo sequence with multiple echo times. Due to the absence of a refocusing pulse, acquisition of T2* mapping is faster than T2 mapping, which facilitates 3D acquisitions. However, it is affected by field inhomogeneity artifacts and, like T2 relaxation, it is prone to the magic angle effect that can potentially lead to misinterpretation of the tissue characteristics or pathology if not properly accounted for [34].

Ultrashort echo time (UTE) enhanced T2* mapping can capture fast-relaxing spins in the deeper and calcified regions of cartilage that are not well-detected by the conventional T2 mapping and may exhibit higher sensitivity to cartilage degeneration [37]. These UTE protocols have echo times about 10–20 times shorter than the shortest echo times generally available on modern clinical MR scanners [38]. The MR signal from highly ordered tissues such as deep and calcified cartilage, cortical bone, and menisci decays very fast so that the echo times used in clinical imaging are unable to produce a signal and so appear dark. The absence of signal from these tissues limits the characterization of their constituents. Williams et al. observed a higher sensitivity of UTE T2* mapping compared to standard T2 mapping in detecting fast relaxing signals within deep cartilage tissue, which implies that UTE T2* mapping potentially could be more effective at capturing specific characteristics or changes in deep cartilage that are not as easily detected by the traditional T2 mapping technique [37]. Liu et al. demonstrated the utility of UTE T2* mapping for visualizing the zone of calcified cartilage in healthy and cadaveric specimens [39]. Moreover, cartilage short T2* components, acquired with UTE acquisition, have been positively correlated with histopathologically confirmed degeneration and alterations in the extracellular matrix organization [40]. However, the advantages of UTE imaging are limited by susceptibility to chemical shift artifacts and off-resonance effects.

The T1 relaxation in a rotating frame (T1ρ relaxation time mapping) has emerged as another valuable method for assessing cartilage quality. In a typical T1ρ relaxation time experiment, following the initial radiofrequency (RF) excitation, the tissue is irradiated with a long, low-amplitude RF pulse, known as the spin-lock pulse. The technique is sensitive to low-frequency macromolecular interactions on the order of the spin-lock pulse frequency [7]. The T1ρ is negatively correlated with the loss of GAG content and variations in fixed-charged density within cartilage tissues [22, 24, 41, 42], which are important indicators of OA-related changes in cartilage. To explain this correlation, it has been suggested that the mechanism dominating T1ρ relaxation in cartilage is the exchange of protons between hydroxyl and amide groups from the GAG chains and bulk water. In early studies, T1ρ has been proposed as a potential alternative for GAG measurement. However, the strength of the correlation between T1ρ and GAG varies with the frequency of the spin-lock pulse, and in the range of frequencies used in biological tissues (< 2 kHz), T1ρ relaxation is also influenced by collagen orientation, and collagen and water content (Fig. 1) [21, 27, 43]. Although the claimed GAG specificity may not hold true, given its relationship with GAG content, tissue hydration, and collagen organization, T1ρ can be used to detect GAG loss, increment in water content and mobility, and loss of cartilage matrix integrity, which are the initial events in the onset of osteoarthritis. Similar to T2, T1ρ values have been shown to increase with cartilage degeneration (Figs. 1, 2) and correlated negatively with cartilage mechanical properties, including equilibrium and dynamic elastic moduli [24, 27, 44]. Typically, multiple images are acquired by varying the duration of the spin-lock RF pulse, and T1ρ values are then estimated by fitting an exponential function to the signal intensity of the images. Despite its potential, T1ρ suffers from similar disadvantages as T2, though the magic angle effect can be mitigated by increasing the spin-lock pulse amplitude. However, this will also lead to increased RF energy deposition within the tissue, which is a concern in T1ρ measurements, especially for in vivo studies as it may result in excessive tissue heating. The RF energy deposition and the magic angle effect can be reduced by employing adiabatic pulses [33].

## GAG-specific methods

**Fig. 3:**
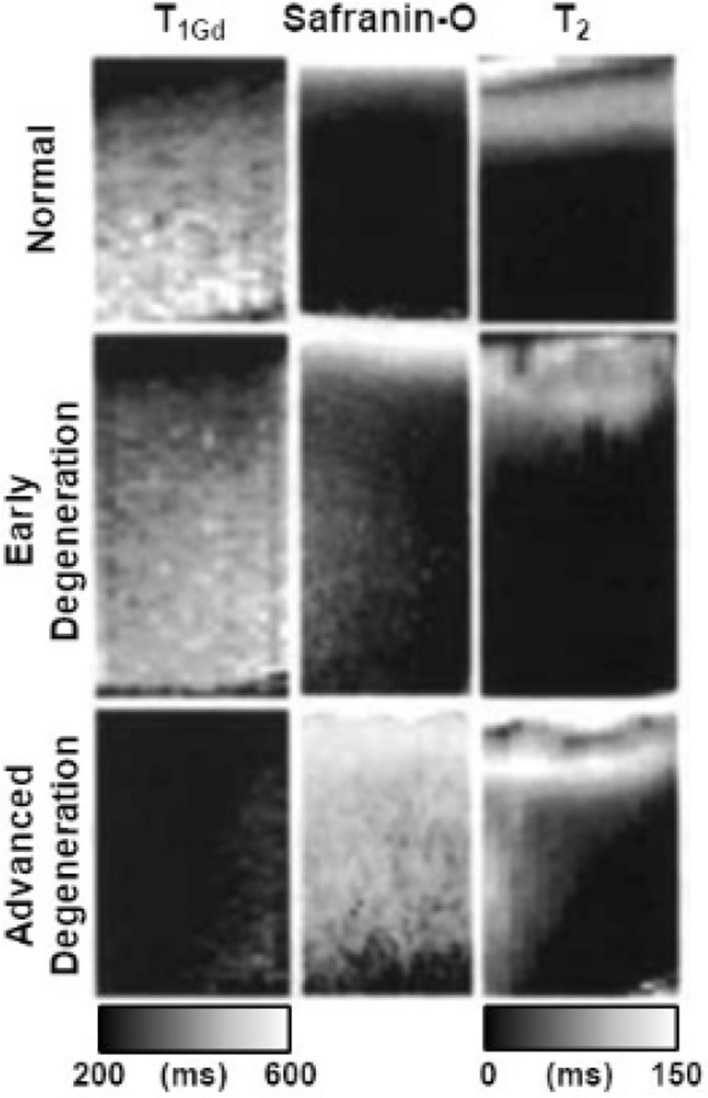
Representative relaxation time maps (dGEMRIC (T1Gd) and T2) and histological image (Safranin-O) of bovine patellar cartilage sample classified according to cartilage quality index (CQI) as normal (CQI = 0–13), early degeneration (CQI = 14–26) and advanced degeneration (CQI = 27–40). The degenerative changes are reflected with decreased T1Gd, increase T2 and confirmed with decreased intensity of Safranin-O. Cartilage surface is towards the top of the figure. Adapted from [22] and reproduced with permission of John Wiley & Sons, copyright 2023

T1 relaxation time mapping has mostly been used in combination with gadolinium-based contrast agents. According to Donnan's theory, in the delayed Gadolinium Enhanced MRI of Cartilage (dGEMRIC) method the negatively charged gadolinium agent accumulates in higher concentrations in areas of lower glycosaminoglycan (GAG) content, which itself contains abundant negatively charged carboxyl and sulfate groups [8]. In these regions, the paramagnetic properties of the contrast agent induce faster T1 relaxation, enabling the use of T1 mapping for spatial assessment of GAG content. The dGEMRIC technique has been validated ex vivo against histological and biochemical measurements of GAG distribution [8, 23] and has demonstrated sensitivity to cartilage degeneration (Fig. 3) [23, 35, 45], as well as positive correlations with cartilage static and dynamic compressive moduli [26, 46, 47]. The technique, however, is not free from uncertainty. Differences in cartilage thickness and permeability can affect contrast agent diffusion, and equilibrium concentrations may not be reached simultaneously in different cartilage zones [48, 49]. Furthermore, dGEMRIC requires long measurement times [50] as T1 mapping must be repeated twice (with and without contrast agent) for accurate GAG content assessment. Pre-contrast T1 mapping is often omitted in clinical studies to reduce scan time. However, this practice may compromise the accuracy of GAG assessment due to variations in native T1 values.

**Fig. 4:**
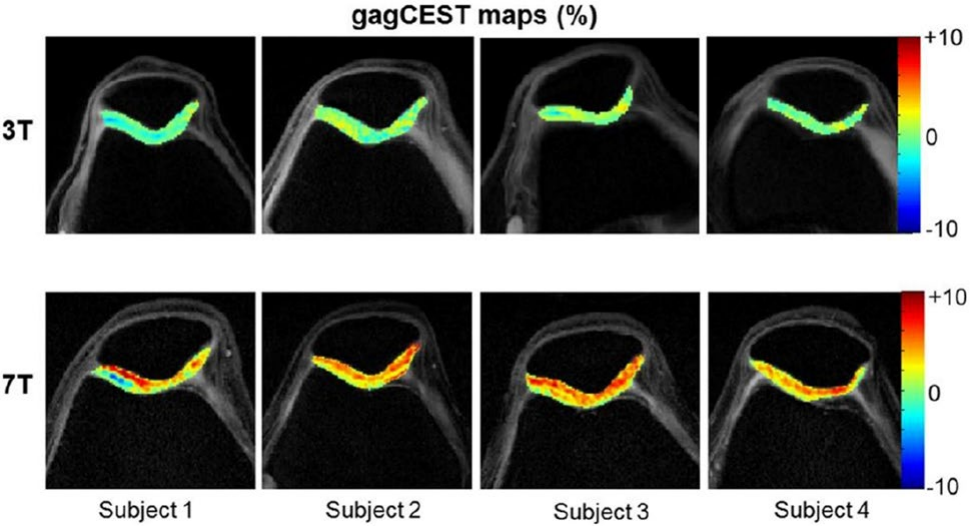
Knee cartilage gagCEST maps from four volunteers at 3 T (top row) and 7 T (bottom row) show improved SNR at higher filed strength. Reproduced from [173] with permission of John Wiley & Sons, copyright 2023

Glycosaminoglycan chemical exchange saturation transfer (gagCEST) is another MRI technique sensitive to the GAG content in cartilage [10]. During a gagCEST experiment, a relatively long and selective RF pulse is applied at specific frequencies to suppress the magnetization of labile protons bound to GAG. As those protons undergo chemical exchange with water molecules, a partial loss in MRI signal intensity is observed. This effect allows for quantification of GAG content in cartilage. The technique benefits from higher magnetic field strengths (> 3.0 Tesla) (Fig. 4) and has been used in OA research. Notably, gagCEST has been demonstrated to detect changes in artificially degraded cartilage [10]; however, validation studies against histological and biochemical GAG content assessments remain limited [51, 52].

**Fig. 5:**
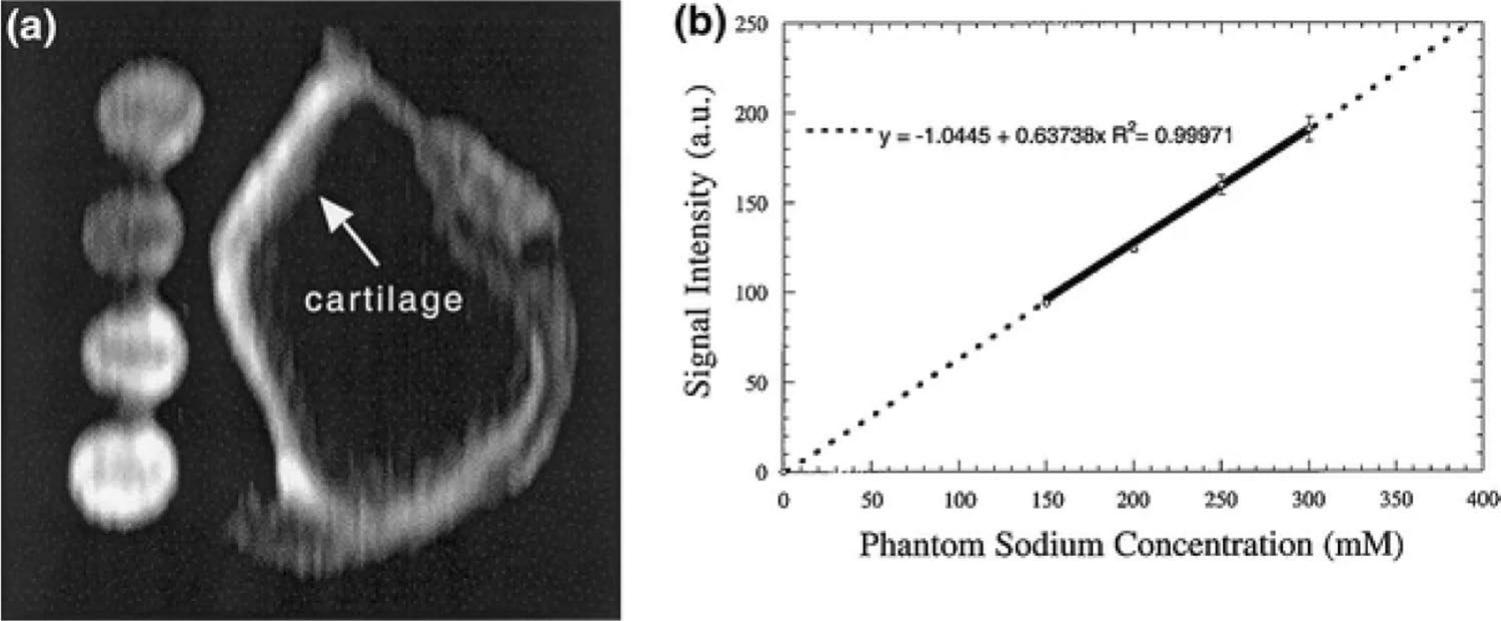
Quantification of sodium concentration in bovine articular cartilage using phantoms as reference. **a** Representative sagittal sodium 3D GRE image of bovine patella and four phantoms with different sodium concentration (from top to bottom: 200, 150, 250, and 300 mM). **b** Curve plot showing the mean calibration obtained from four different slices of a 3D data set. Circles represent signal intensities and error bars indicate the 95% confidence intervals measured from four phantoms. Reproduced from [55] with permission of Elsevier, copyright 2023

Sodium (^23^Na) MRI has been used for the measurement of GAG content in articular cartilage [9]. The negatively charged GAG side chains within the extracellular matrix attract positively charged ^23^Na ions. Under normal physiological condition, these ions distribute proportionally to GAGs, balancing the net charge in cartilage. By assessing sodium concentrations using ^23^Na MRI, fixed charge density in cartilage can be indirectly measured, providing valuable information about GAG content. Absolute quantification of ^23^Na concentration is achieved using reference phantoms with known ^23^Na concentrations (Fig. 5) [53]. This technique directly correlates with GAG content, has demonstrated the ability to differentiate between normal and degenerated cartilage, and has proven to be a useful tool for investigating cartilage maturation [54–56]. The technique has certain limitations due to the low sensitivity of ^23^Na MRI compared to ^1^H MRI. Consequently, sodium MRI has relatively low resolutions (typically on the order of a few millimeters) and requires long measurement times and dedicated hardware, such as RF amplifiers and dedicated coils optimized for signal detection from sodium nuclei.

## Diffusion imaging and quantitative susceptibility mapping of articular cartilage

**Fig. 6:**
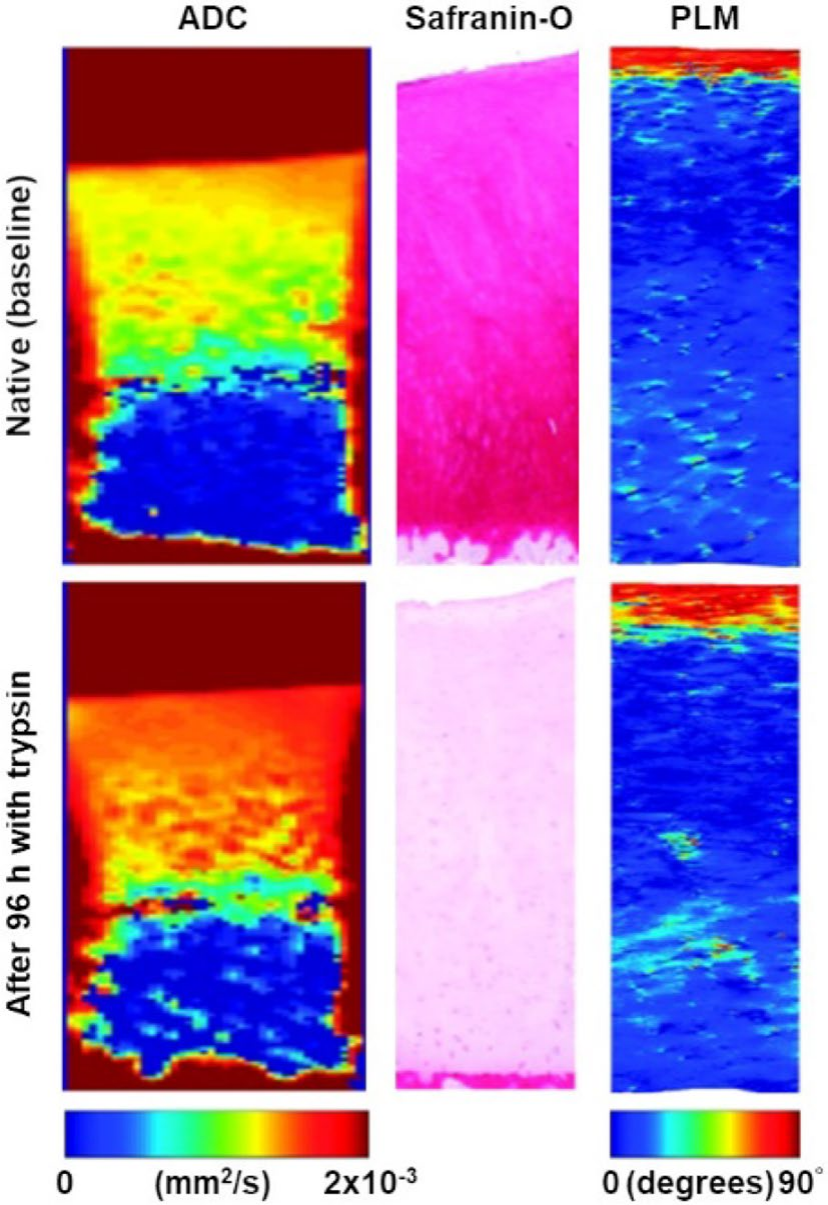
Representative DTI mean diffusivity map (apparent diffusion coefficient, ADC) and histology sections (Safranin-O and PLM) in a sample obtained from a 45-year-old donor. The maps and the histology sections are presented before (top) and 96 h after treatment with trypsin (bottom). Increased ADC values are depicted after the treatment, which are associated with a decrease in intensity of Safranin-O and reflective of progressive PG loss. The treatment did not change the collage architecture as evidenced by PLM. Adapted from [174] and reproduced with permission of Wolters Kluwer Health, Inc., copyright 2023

**Fig. 7:**
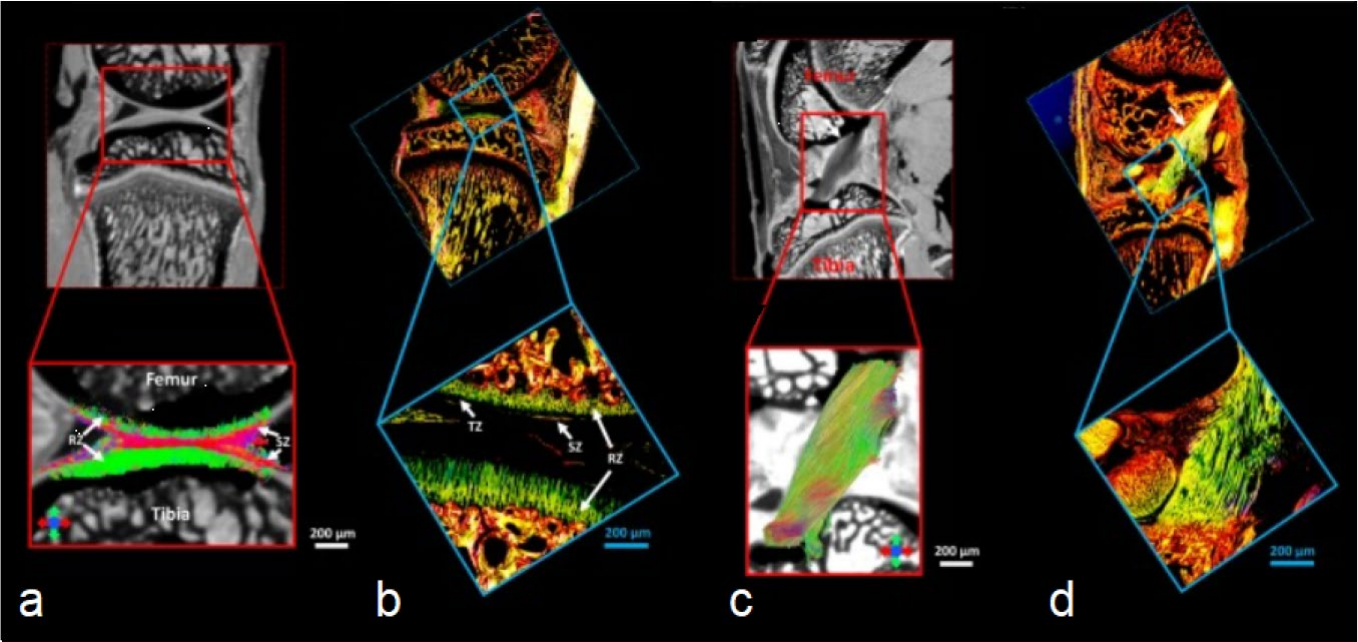
Diffusion MRI tractography (**a**–**c**) and corresponding PLM images (**b**–**d**) of rat joints display collagen fiber orientation in articular cartilage (**a**, **b**) and anterior cruciate ligament (**c**, **d**). Adapted from [61] and reproduced with permission of John Wiley & Sons, copyright 2023

Diffusion-weighted imaging (DWI) utilizes gradient pulses to create contrast based on differences in water motion. Diffusing water molecules differ from degenerated to normal cartilage, where water mobility is restricted by the cartilage components. Consequently, DWI becomes a valuable tool to identify damaged cartilage [57]. Diffusion tensor imaging (DTI) has shown sensitivity to both proteoglycan content, which influences mean diffusivity, and collagen orientation, reflected by fractional anisotropy (Fig. 6) [58]. DTI-based tractography enables visualization of collagen fiber architecture (Fig. 7) [59]. Both DWI and DTI are technically challenging and benefit from higher magnetic field strengths.

Quantitative susceptibility mapping (QSM) is a technique that utilizes MRI magnitude and phase data to estimate the spatial distribution of magnetic susceptibility in tissues [60]. MRI phase data are used to measure field perturbations caused by inhomogeneous susceptibility, which is influenced by tissue properties. Association of QSM with collagen fiber orientation and cartilage calcification [36, 61, 62]. Furthermore, QSM correlates positively with proteoglycan content and cartilage mechanical properties [36]. Notably, QSM has been used to detect abnormalities in cartilage canals in goat model of osteochondritis dissecans [63]. Recently, a study used UTE-QSM for simultaneous quantification of susceptibility properties in articular cartilage and cortical bone [64]. However, further research is required to demonstrate the ability of QSM to detect cartilage degeneration.

## Quantitative methods for menisci, ligaments, and tendons

**Fig. 8:**
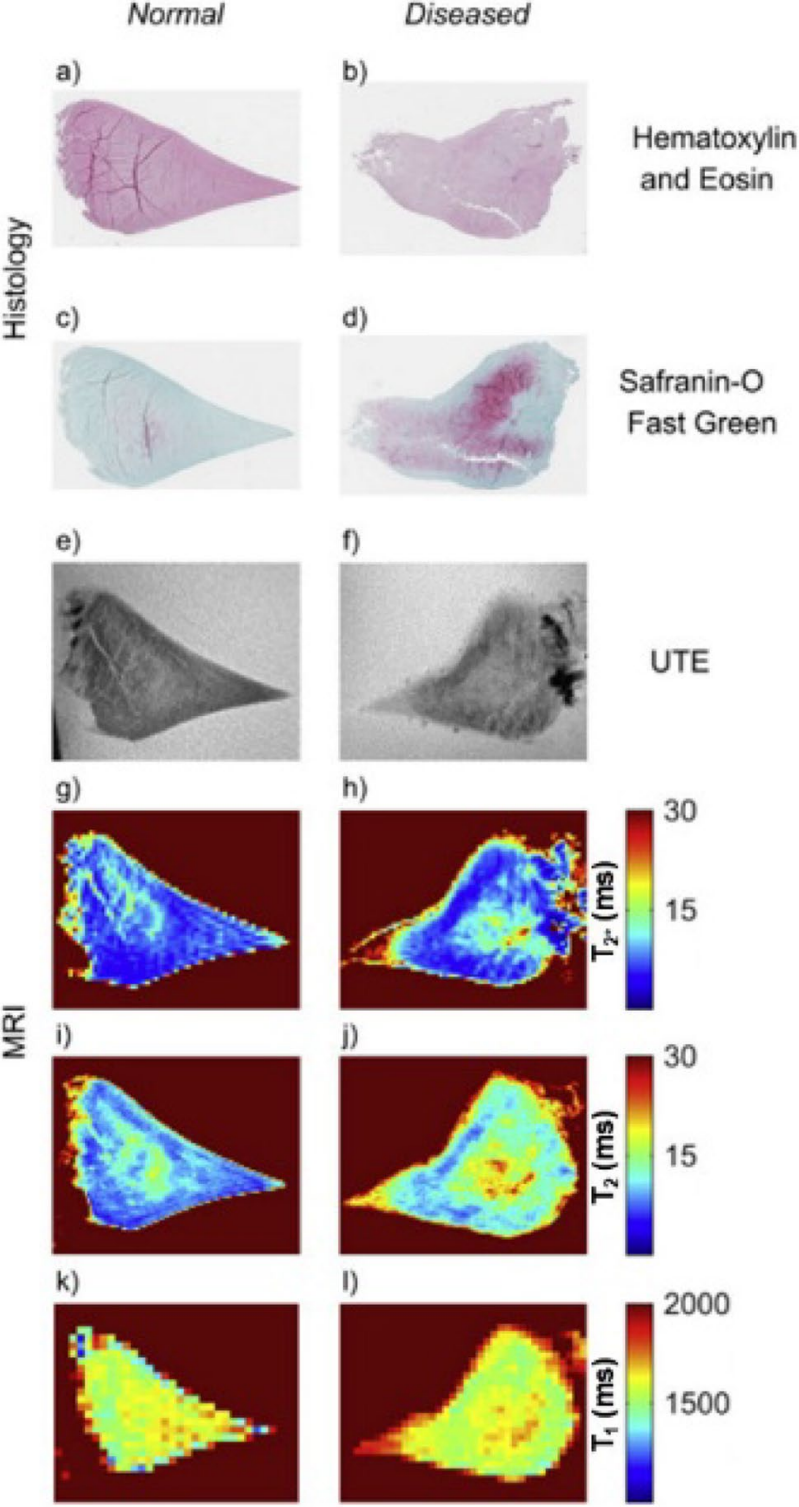
Representative normal and diseased menisci images depicting histology sections (hematoxylin and Eosin (**a**, **b**) and Safranin-O Fast Green (**c**, **d**)), and MRI UTE (**e**, **f**), T2* map (**g**, **h**), T2 map (**i**, **j**) and T1 map (**k**, **l**). Decreased intensity of Safranin-O, reflective of PG content, in the diseased meniscus is associated with increased T1, T2, T2* and UTE. Reproduced from [70] under the terms of the CC-BY Creative Commons Attribution 4.0 International License

Several qMRI techniques used for cartilage have also been applied to assess other cartilaginous tissues. In cases of histologically confirmed meniscal degeneration, elevated T2, T1ρ, and T2* relaxation times have been observed (Fig. 8) [65–69]. Meniscal T2 and T1ρ relaxation times have been associated with water and GAG content, collagen content and organization, and tissue mechanical properties [65, 70]. However, the relationship between these relaxation times and the biochemical content of the meniscus is still not fully understood [65, 68, 70].

For ligament assessment, both T2* and T1ρ have shown negative correlations with ligament elastic modulus [71]. In the case of the Achilles tendon, T2 mapping has been linked to histological evaluation and mechanical properties and has been used to assess tendon healing post-treatment [72, 73]. Additionally, ^23^Na MRI has been shown to positively correlate with GAG content in the Achilles tendon [74].

DTI-based tractography provides visualization of collagen fiber architecture in menisci, tendons and ligaments (Fig. 7) [59, 75, 76]. Furthermore, DTI fractional anisotropy (FA) has been observed to positively correlate with collagen content in the Achilles tendon [73]. DTI has also been used to evaluate the anterior cruciate ligament after reconstruction [77–79] and Achilles tendon after surgical repair [80]. While these findings are promising, more extensive validation of these techniques is warranted.

## Quantitative methods for bones and muscles

**Fig. 9:**
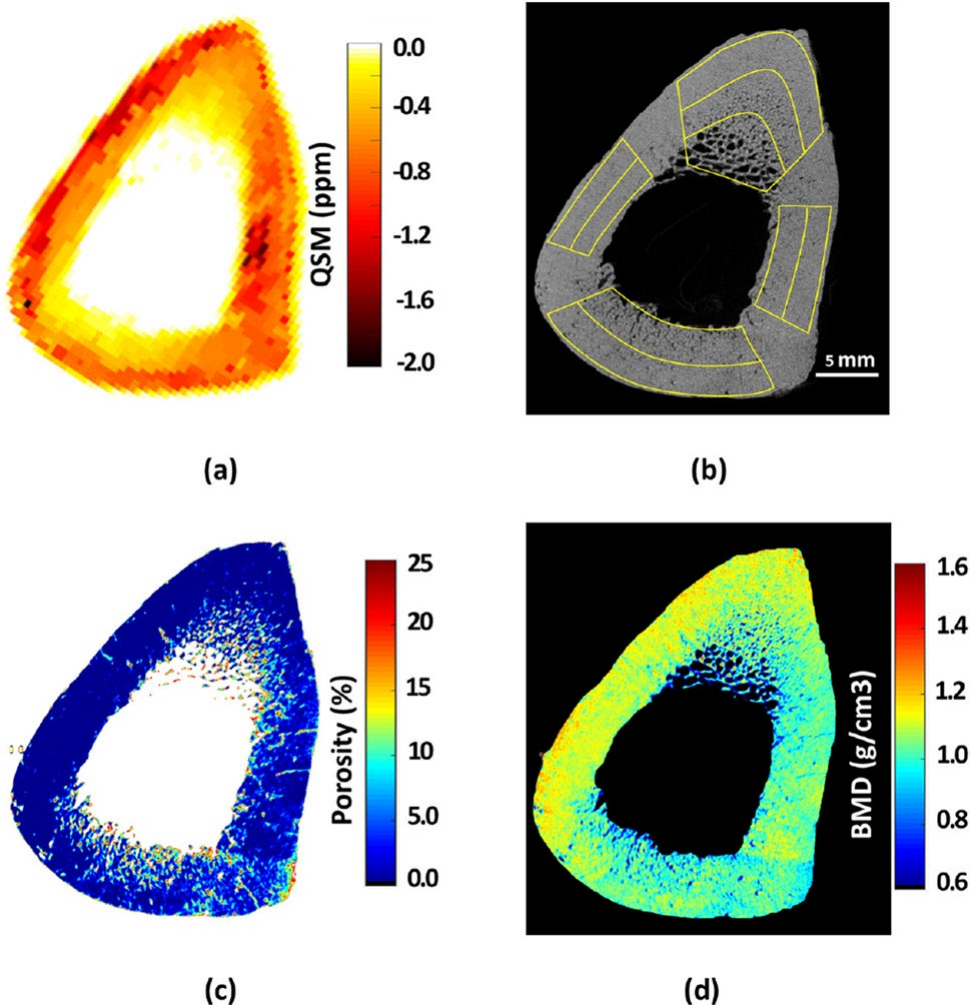
Representative cortical bone sample obtained from tibial midshaft depicts (**a**) QSM, (**b**) uCT slice with the ROIs, (**c**) uCT-based porosity, and (**d**) bone mineral density (BMD) maps. Local maxima as shown in the QSM correspond to the regions of high BMD and low porosity. Reproduced from [91] with permission of Elsevier, copyright 2023

For bone tissue characterization, magnetic field inhomogeneities resulting from susceptibility differences between trabecular and bone marrow are exploited. T2* relaxation time has demonstrated correlations with trabecular bone microstructure, bone mineral density and biomechanical properties [81–84], as well as cortical bone porosity and mechanical properties [85]. Additionally, QSM has been explored for bone characterization [64, 86] and has shown positive correlations with bone porosity and a negative correlation with mineral density (Fig. 9) [87–89].

Fat fraction (FF) MRI techniques can be employed to quantify fat content within bone marrow and skeletal muscles [90–93] and have found significant applications primarily in clinical research related to osteoarthritis, osteoporosis, myopathies, and other metabolic disorders. In addition to FF, various qMRI techniques have been used for the noninvasive assessment of muscular tissues. T2 mapping and ^23^Na MRI have been employed for the characterization of muscle damage [93, 94]. A recent study has shown that FF and T2 mapping are positively correlated with histopathological features in patients with inflammatory myopathies [92]. DTI has been also used to estimate muscle fiber architecture and to visualize microstructural changes due to disease or injury [95, 96]. While these studies have demonstrated the potential of qMRI techniques to identify characteristic features in muscular disorders, their primary application has been in clinical settings. However, research in basic and preclinical domains remains limited, and validation studies are lacking.

## Applications, limitations, and future directions

The primary advantage of utilizing qMRI measurements in studies aimed at advancing our understanding of the underlying biology and pathophysiology of cartilage lies in their noninvasive and nondestructive nature. This characteristic makes qMRI ideal for tracking disease progression over time, unlike histological methods. Another notable advantage of qMRI is the possibility to assess large specimens and whole joints. This can be used for the detection of patterns of regional variability in cartilage degeneration within joints, for instance to identify areas more susceptible to deterioration. Additionally, since it is nondestructive and does not require any special tissue preparation, qMRI is compatible with other techniques. Coupling qMRI with mechanical testing and histology, for example, can aid in understanding the impact of mechanical loading on cartilage integrity, and elucidate how cartilage composition influences its response to mechanical loading in both healthy and degenerated tissue. Quantitative MRI techniques, especially T2, T1ρ, T2*, dGEMRIC, and sodium MRI have been successfully applied to assess cartilage degeneration in both in vivo and ex vivo settings, employing different animal models of OA [27, 55, 97–101]. Moreover, qMRI parameters have been widely used in numerous ex vivo studies to assess the quality and function of human osteochondral samples with varying degrees of degeneration [43, 45, 102–107]. While the strength of the reported correlations varies, T2, T1ρ and T2* were in general sensitive to alterations in collagen fiber network and hydration of the tissue, whereas dGEMRIC and sodium MRI were specific to cartilage compositional changes, and GAG content. These degenerative changes are key indicators of cartilage tissue integrity and reflective of early OA. By understanding the different sensitivities and specificities of these qMRI techniques, researchers can tailor their imaging protocols to comprehensively assess various aspects of cartilage structure, composition, and health.

As for the heterogeneity in the specificity of qMRI parameters reported in the literature, it can be attributed to several factors. First, qMRI measurements are influenced by magnetic field strengths and acquisition protocols, leading to variations in parameter values across different studies. Second, correlations may vary when using cartilage from different animal species or joints due to inherent differences in tissue properties and composition. Furthermore, discrepancies in correlations may arise depending on the reference methods used to assess cartilage constituents. For instance, correlations between T1ρ values and GAG content may differ when using biochemical assays like dimethylmethylene blue compared to optical densitometry with Safranin O staining for GAG quantification [12, 27]. Finally, comparing qMRI data with histology poses challenges due to differences in spatial resolution and tissue processing. MRI slices are typically larger, ranging from hundreds of microns to millimeters, leading to partial volume effects. In contrast, histological sections are typically a few microns thick, with large gaps between slices, and tissue may undergo distortions during processing, resulting in mismatches between the two modalities.

Quantitative MRI techniques have also been instrumental in assessing cartilage restoration after different repair treatments in preclinical animal studies [108–112]. T2 and dGEMRIC have been shown to effectively differentiate repair tissue from native cartilage after microfracture in a goat model and evaluate the regenerative process following allograft chondrocyte implantation in rabbits [108, 110]. Moreover, a significant positive correlation was found between dGEMRIC and GAG concentration in repair tissue [108, 110]. Chu et al. found differences in UTE T2* texture features between chondral defects treated with bone marrow concentrate with and without concomitant microfracture in an equine model [111]. These findings underscore the ability of qMRI to assess structural and biochemical compositional differences following different cartilage repair procedures, providing a noninvasive means for assessing the efficacy of interventions aimed at enhancing cartilage regeneration. Furthermore, qMRI has proven valuable in characterizing engineered cartilage-like tissue [113–118]. For example, in a recent study T2 was used to track the temporal development of fibrocartilage constructs, and it was shown that T2 significantly correlated with their compressive stiffness and coefficient of viscosity [118]. In another study, T2 was demonstrated to correlate with pulsed low-intensity ultrasound-induced changes in cartilage matrix development, indicating its potential for the evaluation of anabolic interventions for engineered tissue [114]. These studies showcase the potential of qMRI in facilitating noninvasive evaluation of engineered cartilage quality in comparison to native tissue, addressing a crucial need in regenerative medicine. In human studies, T2, T1ρ, dGEMRIC and sodium MRI have been applied to evaluate cartilage repair outcomes following bone marrow-stimulating procedures, cartilage grafting techniques, and advanced cell therapy combined with diverse types of biomaterial scaffolds as well as evaluating the quality of regenerated tissue in patients after cartilage repair surgery [14, 109, 119–124]. Another study has also shown the potential of T2 mapping for the assessment and monitoring of meniscal healing status after meniscal repair [125].

Notably, preclinical studies have often used ultra-high magnetic field strengths (≥ 7.0 Tesla) to take advantage of the greater signal-to-noise ratio (SNR) and higher spatial resolution (up to tens of microns) compared to the lower field strengths with submillimeter resolution in clinical studies. Higher spatial resolution and SNR enable visualization of fine anatomical details and enhance the sensitivity of qMRI techniques which are particularly valuable for characterizing small structures or subtle changes in tissue composition and structure. Achieving higher spatial resolution often requires long acquisition times which are more feasible in preclinical or ex vivo studies where logistic constraints are less stringent compared to clinical studies involving human participants. Additionally, ex vivo studies involving tissue specimens can be conducted under controlled conditions coupled with histological analyses. Combining high spatial resolution MRI with histological validation allows for direct correlation between imaging findings and tissue microstructure, which can provide a more thorough understanding of tissue biology, disease processes, and treatment effects.

Ex vivo studies have several limitations. Enzymatic degradation procedures used in those studies are frequently nonspecific to particular components and may also not accurately replicate natural degeneration processes. Moreover, human cartilage samples obtained from joint replacement surgery and cadaveric donors are often scarce, and healthy tissue specimens can be particularly limited. Additionally, certain protocols used in ex vivo studies may not be adaptable for in vivo studies involving human subjects. Therefore, the question remains regarding the generalizability of ex vivo findings to the in vivo setting.

In clinical OA research, qMRI techniques have been widely used in both cross-sectional and longitudinal research studies [4]. Among these techniques, T2 and T1ρ mapping of articular cartilage are the most widely used in clinical studies, as their reliability and discriminative power in identifying OA patients and individuals at risk for OA have been demonstrated [126, 127]. Furthermore, incorporating cartilage T2 mapping in clinical MRI protocols has been shown to enhance sensitivity in detecting cartilage lesions [128]. T2 has been applied to large datasets [129] and used to develop OA diagnostic and prognostic prediction models [130–132]. T2, T2*, T1ρ, and dGEMRIC have also been employed to study cartilage response to mechanical loading in vivo [133–137]. Despite the vast body of evidence supporting their translational potential as biomarkers for OA, qMRI techniques have not yet become routine in clinical practice, primarily due to the lack of standardization in patient preparation protocols, MRI hardware, image acquisition, and processing methods, all of which can impact measurement outcomes [138–143]. To enhance the accuracy of qMRI parameters as biomarkers for monitoring treatment response, further basic research is warranted to gain an in-depth mechanistic understanding of the relationship between biological processes and qMRI. Additionally, it is essential to verify how well the findings obtained at higher magnetic field translate to clinical settings. Therefore, rigorous validation studies are needed to assess the reproducibility and reliability of qMRI measurements across different MRI platforms, acquisition parameters, and patient populations. This will ensure that qMRI biomarkers retain their diagnostic and prognostic value in clinical practice and enable clinicians to make informed treatment decisions based on qMRI-derived information.

The integration of artificial intelligence (AI) tools, particularly deep learning algorithms, may help reduce variability in image acquisition and analysis in MRI studies, including those focused on assessing cartilage health [144, 145]. AI-driven automated segmentation algorithms can accurately delineate cartilage boundaries and automated segmentations of cartilage structures have been reported to closely agree with those performed by expert radiologists and clinicians [144]. These algorithms can learn complex patterns and features from large datasets, enabling them to adapt to variations in imaging protocols and scanner characteristics. By automating the segmentation process, AI tools help ensure standardized and reproducible measurements across different MR scanners, acquisition parameters and subject population; thereby reducing variability and enhancing the reliability and comparability of MRI data obtained from different sources. In the future, deep learning may play a crucial role in image reconstruction to mitigate image acquisition variability. However, this aspect has yet to be explored, as to date deep learning-based image reconstruction algorithms have been applied to shorten scan times in clinical settings [146, 147].

Texture-based radiomics analysis of qMRI has emerged as a valuable approach for detecting changes in the distribution of the cartilage matrix associated with degeneration. The gray-level co-occurrence matrix (GLCM) allows for the extraction of statistical measures of the spatial distribution of pixel values [148], aiding in the identification of textural differences between healthy and pathological tissue, which exhibit significant spatial variation in cartilage relaxation time values compared with normal cartilage [149]. Notably, GLCM texture features derived from cartilage T2 and T1ρ maps have shown enhanced discriminative power in detecting early structural changes [149–156]. Recently, GLCM texture analysis of T2 maps has been used to assess repair tissue and tissue adjacent to the repair site after matrix-associated chondrocyte transplantation [124, 157]. While GLCM-based texture features are commonly used in the context of OA research, local binary pattern-based texture analysis [158] of T2 maps was explored in one study and demonstrated significant differences between OA vs asymptomatic knees [159]. Texture analysis holds the potential to boost qMRI sensitivity to pathological changes in cartilage; however, further validation in basic studies is necessary, and it remains unknown which specific biochemical changes are reflected in different textural features. Furthermore, the reproducibility of texture analysis of qMRI has not been systematically studied and is reliant on factors such as image resolution, acquisition sequence, imaging parameters, and selection of input parameters (e.g., orientation, inter-pixel distance, number of gray levels), as well as the data preprocessing pipeline used.

**Fig. 10:**
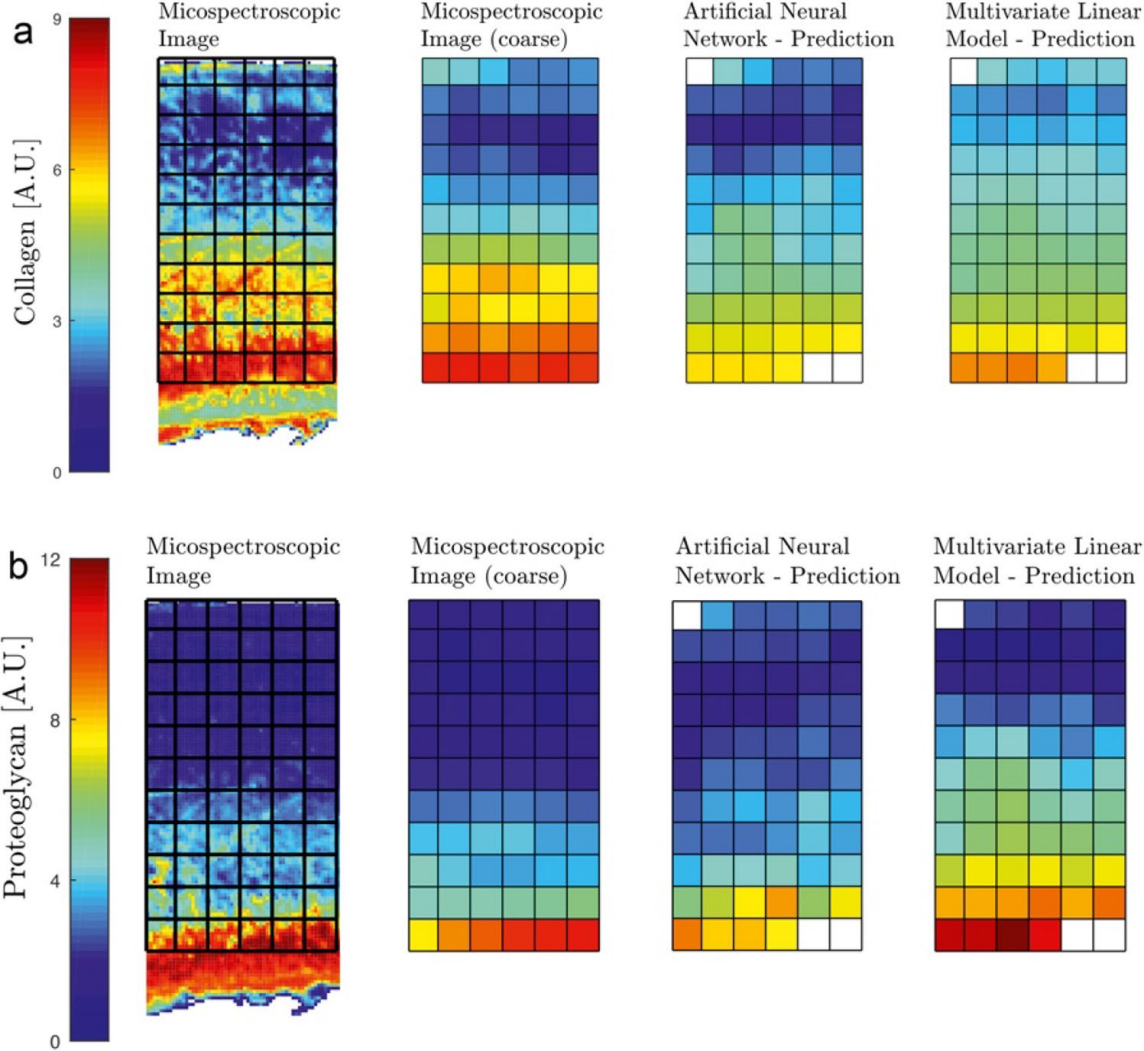
Spatial distributions of collagen (**a**) and proteoglycan content (**b**) in a human cartilage specimen: spectroscopic measurements at original resolution (first column) and after downsampling to match MRI resolution (second column); multiparametric qMRI-based predictions with artificial neural network (third column), and multivariate linear model (fourth column). Reproduced from [170] with permission of Elsevier, copyright 2023

Advancing the sensitivity to degeneration and specificity to cartilage structural properties and biochemical components is achievable through the combination of multiple qMRI techniques [116, 160–162]. Recent studies have utilized several qMRI parameters in conjunction with machine learning techniques to estimate proteoglycan and collagen content (Fig. 10) and collagen fiber orientation in cartilage specimens [163, 164]. The findings demonstrated high prediction accuracy and moderate-to-strong correlations between predicted and measured cartilage compositional and structural parameters. These findings hold great promise for future in vivo applications. However, the clinical translation of tools based on measurements of multiple qMRI parameters is complicated by the relatively long acquisition times of conventional sequences for quantitative cartilage mapping. This limitation could potentially be overcome by novel methods enabling rapid measurements of multiple qMRI parameters, such as magnetic resonance fingerprinting [30, 165, 166].

In conclusion, qMRI techniques have extended the capabilities of MRI beyond traditional morphological imaging, enabling indirect assessment of cartilage microstructure, biochemical content, and function. Several of these techniques have been extensively validated in cartilage and provide valuable tools in basic and translational research for a deeper understanding of disease mechanisms, cartilage degeneration and repair processes, and for characterizing engineered cartilage-like tissue. Moreover, qMRI holds significant promise in contributing to the development of innovative and novel therapeutic approaches for cartilage-related disorders. The non-destructive nature of qMRI allows for repeated measurements over time, facilitating longitudinal studies to track disease progression and monitor therapeutic interventions.

While T2 and T1ρ mapping lack specificity to biochemical components of cartilage, they have demonstrated the ability to assess the structural integrity of the extracellular matrix and showed their potential in OA diagnostics. On the other hand, promising techniques auch as DTI, sodium MRI, and gagCEST, though presenting technical challenges and requiring higher magnetic field strengths, hold significant potential for precisely assessing cartilage changes in osteoarthritis.

The current evidence supporting qMRI's value in menisci, tendons, and ligaments is relatively limited, necessitating further investigation and validation. Standardization of measurements and analysis protocols remains a challenge. Addressing these challenges, potentially through the integration of artificial intelligence, will be crucial in realizing the full potential of qMRI in clinical practice. Further basic research to better understand the underlying biological processes is an essential step toward enhancing the accuracy and applicability of qMRI parameters as biomarkers for monitoring treatment response.

Texture-based radiomics and combining multiple qMRI techniques offer promising avenues for improving the sensitivity to degeneration and specificity of cartilage structural properties and biochemical components. These approaches hold great potential for providing a more comprehensive and detailed assessment of cartilage, and they are poised to play an increasingly significant role in cartilage research and potentially transforming the diagnosis and treatment of cartilage-related disorders.

## Data Availability

Not applicable, this is a review paper as such and not involving any data collection/analysis.
